# Microwave Ablation of Pulmonary Carcinoid Tumor: A Therapeutic Alternative for Inoperable Cases

**DOI:** 10.5334/jbsr.3960

**Published:** 2025-08-14

**Authors:** Thierry Nzoyisaba, Jean-François Goyers

**Affiliations:** 1 CHU de Liège, Avenue de l’Hôpital 1, 4000 Liège, Belgium; 2 CHC MontLégia, Boulevard Patience et Beaujonc 2, 4000 Liège, Belgium

**Keywords:** pulmonary carcinoid tumor, ACTH‑dependent Cushing’s syndrome, microwave ablation, thermal ablation, minimally invasive treatment, inoperable lung tumor

## Abstract

Pulmonary carcinoid tumors are rare and may lead to severe paraneoplastic syndromes. A case is reported of an inoperable 78‑year‑old patient with ACTH‑dependent Cushing’s syndrome successfully treated with microwave ablation. The procedure resulted in rapid clinical improvement without complications and radiological stability.

*Teaching point:* The role of microwave ablation as a minimally invasive alternative for selected inoperable patients, offering an effective tumor control strategy with minimal morbidity.

## Introduction

Pulmonary carcinoid tumors (PCTs) are rare neuroendocrine neoplasms, accounting for 1–2% of all lung cancers [1]. They are classified as typical or atypical, with the latter being more aggressive [2]. Surgical resection is the standard treatment, but many patients, particularly the elderly or those with significant comorbidities, are not eligible for surgery [3]. Image‑guided thermal ablation techniques have emerged as alternative local therapies.

Microwave ablation (MWA) has demonstrated promising efficacy in early‑stage non‑small cell lung cancer (NSCLC), particularly in ground‑glass opacity lesions [3, 4]. Compared to radiofrequency ablation (RFA), MWA achieves higher temperatures, larger ablation zones, and shorter procedure times, with reduced heat‑sink effects [4]. Although data on MWA in PCTs are limited, early reports suggest it may offer a viable option in selected inoperable patients [1]. A case of ACTH‑secreting pulmonary carcinoid tumor successfully treated with MWA is reported.

## Case Report

A 78‑year‑old woman was referred for management of a pulmonary carcinoid tumor responsible for ACTH‑dependent Cushing’s syndrome. She presented with severe weight loss, newly diagnosed diabetes, and cognitive decline. Medical history included heart failure with pacemaker implantation, treated breast cancer, and chronic atypical mycobacterial infection. Functional status was poor, with significant muscle wasting and a 10 kg weight loss over the last three months.

**Figure 1:**
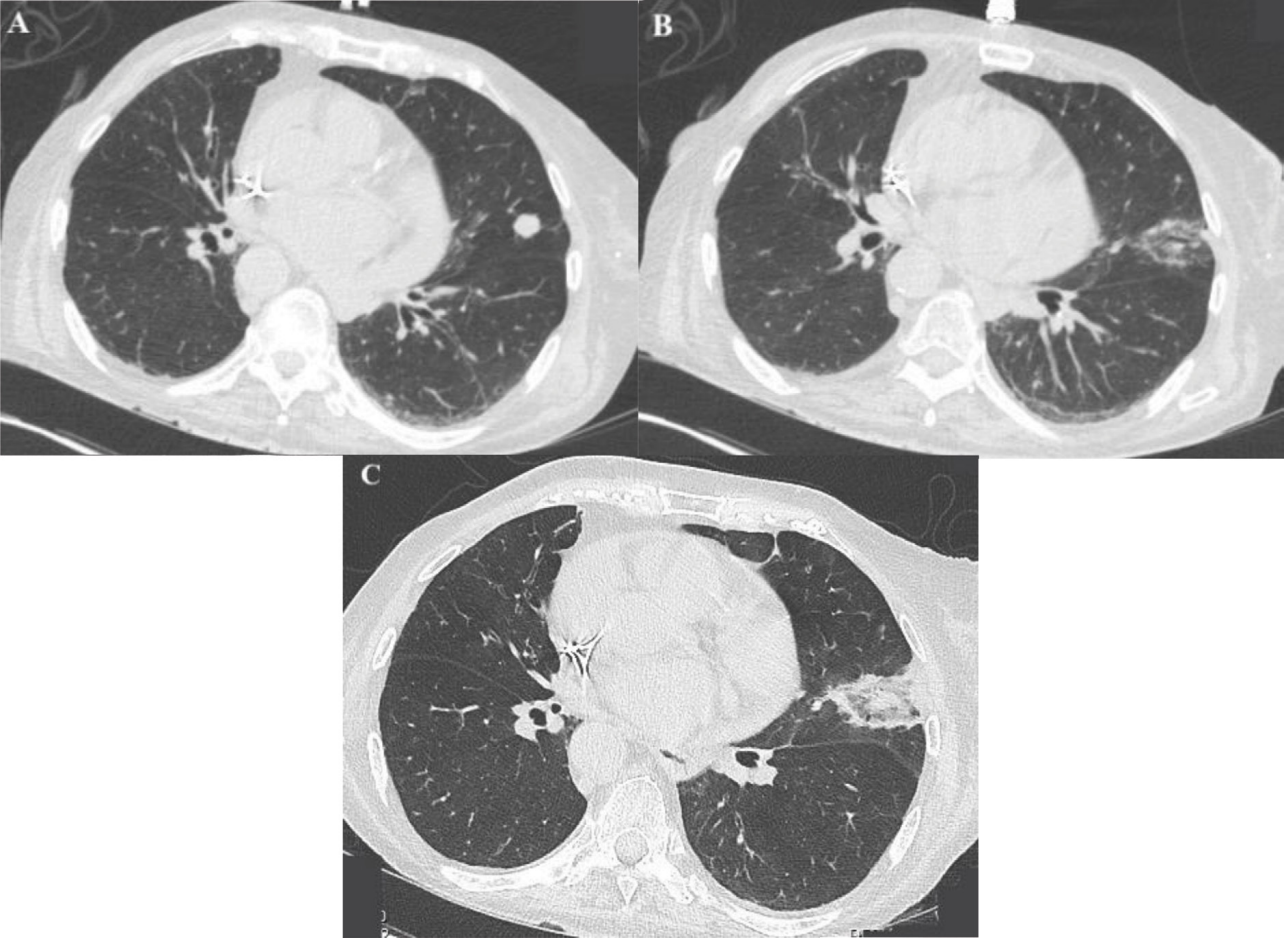
Axial CT images of the pulmonary carcinoid tumor during the ablation procedure before and after **(A** and **B)**, and on post‑procedural day 1 **(C)**, demonstrating ablation zone and absence of immediate complications.

Hormonal investigations confirmed an ACTH‑dependent Cushing’s syndrome. PET‑CT identified a hypermetabolic 1.5 cm nodule in the left upper lobe, corresponding to a well‑differentiated carcinoid tumor on transthoracic biopsy (Ki‑67 <2%). Given the patient’s high surgical risk, percutaneous MWA under general anesthesia was performed. The procedure was technically successful and complication‑free (Figure 1). The patient was discharged the following day.

**Figure 2:**
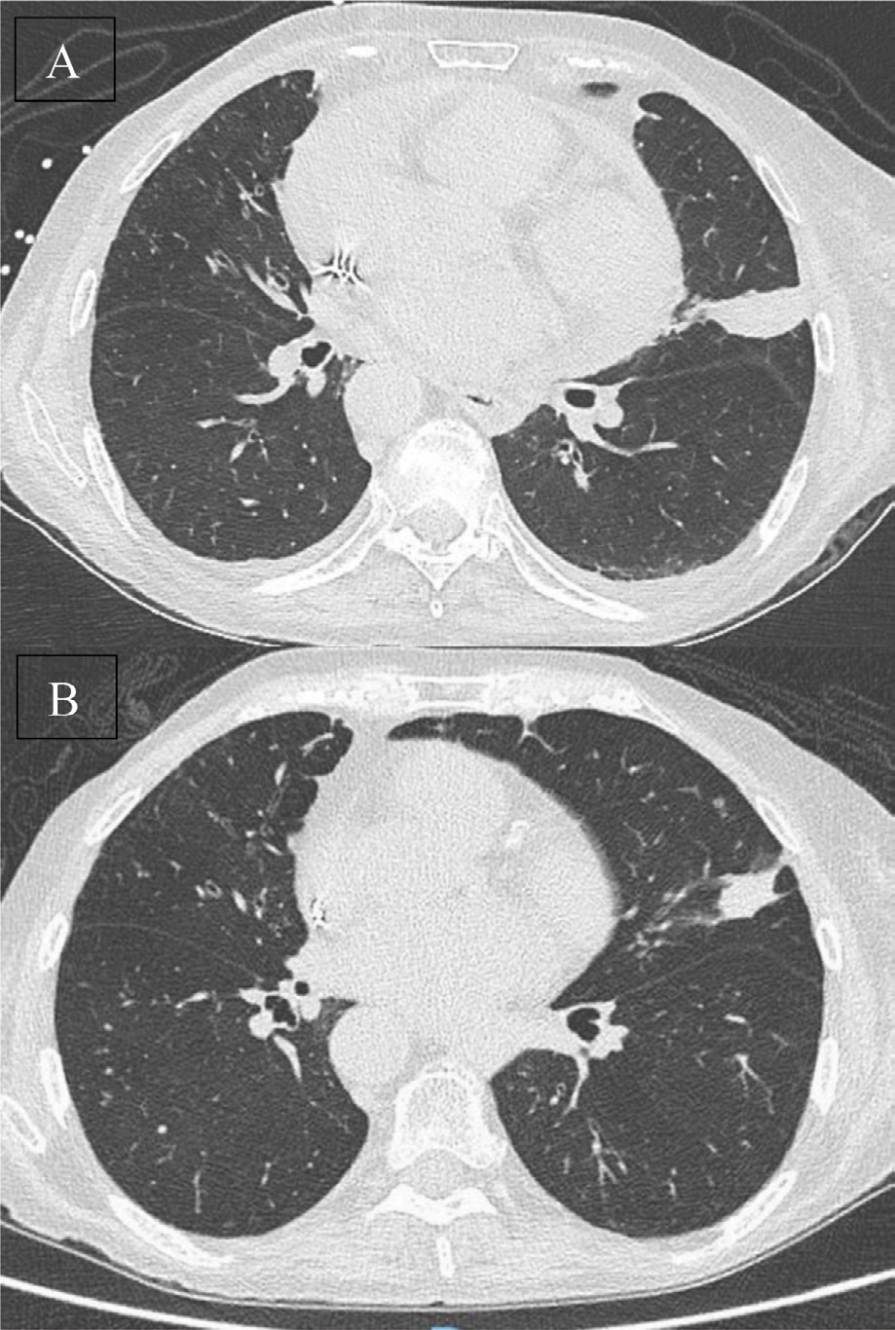
Axial CT images of the treated pulmonary carcinoid tumor at 1‑month **(A)** and 5‑month **(B)** follow‑up, showing sustained regression of the ablation zone without evidence of recurrence or new complications.

A five‑month follow‑up CT showed a partial regression of the post‑treatment consolidation compared to the one‑month control, without signs of lesion progression (Figure 2). Glycemic control improved markedly, and signs of cortisol excess, including sarcopenia and cognitive disturbances, stabilized.

## Discussion

Surgical resection remains the gold standard for PCTs, offering five‑year survival rates exceeding 90% in typical forms [2]. However, surgery is contraindicated in patients with advanced age or comorbidities. In such cases, minimally invasive techniques like thermal ablation may be considered [1].

MWA induces coagulative necrosis via high‑frequency electromagnetic energy, causing rapid molecular oscillation and heat production. Compared to RFA, MWA produces higher and more uniform temperatures, enabling effective ablation of larger tumors in shorter times. It is also less susceptible to heat‑sink effects near blood vessels [5]. These features make MWA particularly attractive for lung tumors.

Although most evidence for MWA comes from NSCLC, retrospective studies and SEER database analyses reported that thermal ablation improves both overall and cancer‑specific survival compared to no intervention [1]. MWA appears most effective in tumors ≤3.5 cm, with local control rates reaching 96% at one year [5]. This patient’s tumor was consistent with these criteria, and the post‑procedure imaging confirmed local control at three months.

The safety profile of MWA is generally favorable. The most common complication is pneumothorax (8.5–63%), though only a minority require drainage [5]. Other adverse events include periprocedural pain, pleural effusion, hemoptysis, and rare infections or bronchopleural fistulas [5, 6]. In this case, the procedure was entirely complication‑free, with no adverse events observed during or after the intervention.

The case adds to the growing literature supporting MWA as a feasible alternative for inoperable pulmonary carcinoid tumors. It is especially relevant in hormone‑secreting tumors, where rapid symptom control is vital. In our patient, MWA contributed to stabilization of hypercortisolism and metabolic parameters.

## Conclusion

MWA represents a promising alternative in selected patients with inoperable pulmonary carcinoid tumors. In this case, MWA resulted in symptomatic improvement and radiological stability without complications. Although surgery remains the standard, MWA offers a minimally invasive option for patients unfit for resection. Further prospective studies are needed to validate its efficacy and define its optimal role.
